# Amyand’s Hernia: A Snap From the Learned Mind

**DOI:** 10.7759/cureus.69948

**Published:** 2024-09-22

**Authors:** Bikram Rout, Harshad Murgaonkar, Ankur Cheleng, Srikanth Chinthala

**Affiliations:** 1 General Surgery, All India Institute of Medical Sciences, Bhubaneswar, Bhubaneswar, IND

**Keywords:** amyand’s hernia, emergency appendectomy, incarcerated inguinal hernia, strangulated inguinal hernia, the appendix

## Abstract

Amyand’s hernia is a rare clinical condition containing appendix as the content of hernia. The incidence of this type of hernia is rare; the appendix may become incarcerated within the sac and can lead to strangulation or perforation. This case report highlights the clinical presentation, diagnosis, and management of an Amyand’s hernia in a strangulated masquerade in a 75-year-old male. It signifies the importance of early recognition and prompt surgical intervention to counteract potential complications associated with this condition.

## Introduction

Amyand’s hernia is a clinical condition with vermiform appendix being the content of the hernial sac. It is an extremely rare clinical condition with 1% incidence and 0.4% expected complications [1]. An Amyand's hernia has the potential to become inflamed, infected, or perforated, with the possibility of the appendix being incarcerated, while in some instances, the content may remain healthy. The vague clinical manifestations and a lack of distinct radiological diagnostic characteristics make it difficult to make a definite preoperative diagnosis [2]. Furthermore, the scant literature on the subject poses a challenge for physicians seeking to learn about Amyand's hernia and incarcerated appendices.

Here, we are discussing a similar case with an inflamed appendix diagnosed intraoperatively, followed by prompt and appropriate steps taken in its management. The aim of this case report is to present new perspectives on the diagnosis and treatment of Amyand’s hernia and its associated complications.

## Case presentation

A 75-year-old male presented to the emergency room with complaints of pain and swelling in the right inguinoscrotal region for the last four days. The swelling was initially reducible over the past one year and was associated with irreducibility, pain, and redness over the last four days. There was no fever or symptoms pertaining to obstipation. On examination, patient was conscious, oriented, and well built with signs of dehydration and tachycardia. The inguinoscrotal swelling was 10 cm x 5 cm extending to the base of the scrotum with redness over the swelling and tenderness on palpation and was irreducible. Abdomen and per rectal findings were within normal limits with audible bowel sounds. A clinical diagnosis of strangulated inguinal hernia was established. Ultrasonography also gave a similar diagnosis. The patient's hemoglobin level was 9.0 gm/dL, leukocyte count was 12,200 ug/dl, and the patient was taken for inguinal exploration in the emergency theater (Table 1).

**Table 1:** Lab test results on admission *as per the reference range values used in the institution (AIIMS, Bhubaneswar)

Investigations	Result	Unit	Ref. range*
Hemoglobin	9.0	gm/dL	13-17
Total leukocyte count	12.2	*10^3^/cumm	4-11

**Figure 1:**
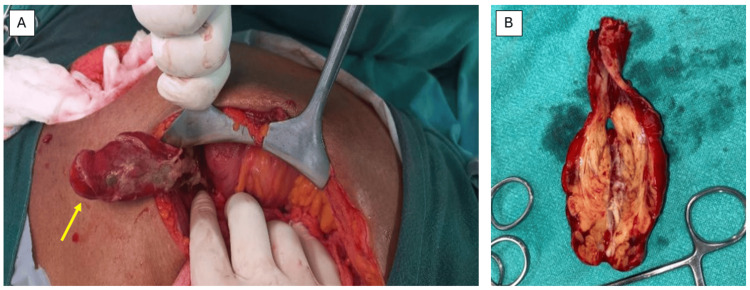
Intraoperative findings. (A) Inflamed and thickened appendix as the content of hernial sac. (B) Cut section of the resected appendectomy specimen

On inguinal exploration, a thick tubular inflamed structure as the content was found which was adherent to the sac. Due to difficulty in identifying the content after opening the sac and associated difficult retrograde tracing as a result of constricted deep ring opening, a low midline laparotomy was done upon which the tubular structure was seen to be in continuity with the cecum, which was partly inflamed along with the ileocecal junction. Hence, an intraoperative diagnosis of Amyand’s hernia was made, with an inflamed appendix of length 12 cm and a diameter of 15 mm (Figures 1A-1B). An appendectomy was performed followed by Bassini’s anatomical repair for the right inguinal hernia. The laparotomy wound was closed, and the specimen was sent for histopathology.

**Figure 2:**
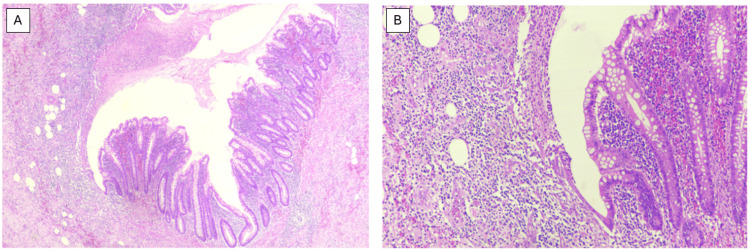
(A) Hematoxylin and eosin staining (40x magnification). (B) Hematoxylin and eosin staining (100x magnification) of excised appendix with inflamed wall

Postoperatively, the patient was started orally after 48 hours, with mild serous discharge from the right groin surgical wound on day three managed conservatively with daily dressing. The patient was discharged on postoperative day four in a hemodynamically stable state. On follow-up, wound was healthy. Histopathology confirmed the excised specimen as an appendix with an inflamed wall (Figures 2A-2B).

## Discussion

Claudius Amyand in 1735 operated on a case of right inguinal hernia with fecal discharging fistula in the groin, which by tracing with a pin led to the discovery of the “vermiform appendix” as the content. The surgeon excised it and repaired the hernia for which it was bestowed the title “Amyand’s hernia” a century later [3]. The patent processus vaginalis makes the condition more common in adults [4]. In cases of malrotation of the gut, Amyand’s hernia typically presents on the left side; however, the cases of malrotation of the gut being rare, the Amyand’s hernia is found to occur mostly on the right side [5].

It remains a clinical challenge to diagnose this condition on mere bedside examination, as its features mirror those of inguinal hernia ranging from simply being asymptomatic [6], to reducible unless it is incarcerated or strangulated. Here, a radiological aid bears capacity for prospective diagnosis of such a rare condition [7,8].

Amyand’s hernia has been classified into four different types by Losanoff and Basson in the following manner along with the proposed surgical treatment for each of its types (Table 2) [9,10].

**Table 2:** Amyand's hernia classification by Losanoff and Basson

Classification	Description	Surgical management
Type I	Noninflamed appendix as content within the inguinal region	Hernia reduction, mesh repair
Type II	Inflamed appendix as content within inguinal region	Hernia reduction, appendectomy, primary repair of hernia
Type III	Acute appendicitis within inguinal region, complicated peritonitis or abdominal wall sepsis	Hernia reduction, appendectomy, primary repair of hernia, +/- laparotomy
Type IV	Acute appendicitis within inguinal region, related or unrelated abdominal pathology	Treated as type II to III + investigate or treat other pathology appropriately

In 2011, Singal and Gupta proposed an updated classification system, taking into account the possibility of an incisional hernia resulting from a previous abdominal incision. As a result, type V, an additional subtype, which was further divided into three categories, was proposed. A normal appendix within an incisional hernia was defined as subtype VA and which was to be managed as type I. Subtype VB pertained to appendicitis trapped within the sac of an incisional hernia, necessitating treatment with appendectomy followed by mesh hernioplasty repair and subtype VC referred to appendicitis within the sac of an incisional hernia alongside another abdominal condition, requiring a management approach akin to that of type IV [11].

The most widely done primary repair was Bassini’s repair [12,13]. All cases of uncomplicated appendix within the hernial sac do not mandate an appendectomy [14,15]. However, most surgeons do an appendectomy in children as it may re-herniate or if it is of the left side and a mobile cecum is enough to herniate again [16,17]. In a minimal invasive approach, delicate retraction of the appendix through the hernial defect is done, and the same principles as mentioned above are applied [18].

The complications include acute appendicitis, appendicular perforation, perforation peritonitis, peri-appendicular abscess, necrotizing fasciitis [19], strangulation [20], testicular ischemia in newborns resulting from cord compression, and rarely, malignancy. Some studies also justify preserving an uncomplicated appendix for its immunological properties. The patient here was diagnosed and treated as type II Amyand’s hernia according to the Losanoff and Basson classification.

The need for a laparotomy in every case of Amyand's hernia is also debatable. Types III and IV are generally associated with more chances of a midline laparotomy. The difficulty to define the content of the sac during inguinal exploration due to adhesions and inflammation, the presence of perforation and contamination with signs of peritonitis preoperatively, lead to more chances of a midline exploration.

## Conclusions

Although rare, acute appendicitis and its following dreaded complications as Amyand’s hernia need prompt intervention, as this is usually diagnosed incidentally either via imaging or more frequently intraoperatively. A clinical vigilance of a possible Amyand’s hernia is to be kept as a differential where it masquerades around as an inguinal hernia, especially in children. In our case, Amyand’s hernia was identified and operated on before it could progress to perforation and possible abscess and peritonitis. We have tried to provide another perspective on the existing classification of Amyand's hernia by associating the degree of severity with the increase in the chances of a midline laparotomy vs inguinal exploration. The need for a laparotomy increases with increasing complications of Amyand's hernia. 
